# Apophysenschäden im Sport

**DOI:** 10.1007/s00132-021-04074-z

**Published:** 2021-01-29

**Authors:** Markus Neubauer, Stefan Nehrer

**Affiliations:** 1 Zentrum für Regenerative Medizin, Donau Universität Krems, Dr. Karl-Dorrek-Str. 30, 3500 Krems, Österreich; 2 Universitätsklinik für Orthopädie, Universitätsklinikum Krems, Karl Landsteiner Privatuniversität für Gesundheitswissenschaften, Dr. Karl-Dorrek-Straße 30, 3500 Krems, Österreich

**Keywords:** Hochintensives Training, Systematische Trainingsplanung, Mechanische Überbeanspruchung, Wachstumsalter, Bildgebung, High-intensity training, Systematic training plan, Mechanical overstraining, Growth period, Imaging

## Abstract

Die Zahl an Jugendlichen und Kindern im Spitzen- oder hochintensiven Breitensport ist – bezogen auf Industrienationen – im Steigen begriffen. Hochintensives Training kann Überlastungen durch die vermehrte Zugwirkung besonders auf Sehnen- und Muskelinsertionsstellen bedingen. Apophysen als Ossifikationskerne in Sehnen- und Muskelinsertionen sind bei Heranwachsenden besonders vulnerabel für überlastungsbedingte Pathologien. Zentrale Maßnahmen in der Prävention sind eine systematische Trainingsplanung und die Vermeidung mechanischer Überbeanspruchung im Wachstumsalter. Eine exakte Bildgebung ermöglicht die Diagnose von Frühstadien apophysärer Strukturschäden, die in dieser Phase durch Trainingspause und konservative Maßnahmen geheilt werden können.

## Abstract (en)

The number of adolescents and children in elite and high-intensity mass sports is increasing, with respect to industrial nations. High-intensity training can cause overload due to the increased traction effect, particularly on tendon and muscle insertion sites. Apophyses are the center for ossification in tendon and muscle insertions and are therefore particularly vulnerable in youths to overload-related pathologies. Core measures in the prevention are a systematic planning of training and the avoidance of mechanical overstraining in the growth period. An exact imaging enables the diagnosis of apophyseal structural damage at an early stage, which in this phase can be healed by a pause in training and conservative measures.

## Lernziele

Nach Lektüre dieses Beitrags …

- können Sie 3 der häufigsten Apophysenkrankheitsbilder und deren Lokalisation an der unteren und oberen Extremität benennen,
- verstehen Sie den zugrunde liegenden Pathomechanismus als Locus minoris resistentiae,
- haben Sie Kenntnis der gängigen Diagnostik und Therapieindikationen.

## Einleitung

Die Zahl an Jugendlichen und Kindern im Spitzen- oder hochintensiven Breitensport ist – bezogen auf Industrienationen – im Steigen begriffen [1, 2]. Diese per se erfreuliche Entwicklung führt aber auch zu einer Erhöhung der **sportassoziierten Verletzungen**

sportassoziierten Verletzungen.

Dieser Umstand erfordert daher immer mehr die Integration eines zielorientierten und professionell ausgearbeiteten Trainings im Kindes- und Jugendalter, um den besonderen Anforderungen des wachsenden muskuloskeletalen Systems in dieser Phase erhöhter Vulnerabilität gerecht zu werden. Denn der aktive und passive Bewegungsapparat wird hier in der Phase der Entwicklung und des Wachstums gefordert. Die aktuelle Literatur zeigt für regelmäßiges, moderates Training einen positiven Effekt auf das psychophysische Gedeihen von Kindern und Jugendlichen [3, 4]. Ein wachsender „body of evidence“ zeigt demgegenüber für hochintensives Training junger Athleten über lange Zeit negative Effekte [1, 5, 6, 7, 8]. Insgesamt muss jedoch darauf hingewiesen werden, dass die meisten Daten zum Thema der apophysären Verletzungen aus Arbeiten stammen, die nicht das höchste Evidenzniveau aufweisen wie Single-Case-Reports oder Case-Series, und somit vermehrt randomisiert kontrollierte Studien zu assoziierten Fragestellungen wünschenswert und notwendig sind [9].

**Hochintensives Training**

Hochintensives Training

kann Überlastungen durch die vermehrte Zugwirkung besonders auf Sehnen- und Muskelinsertionsstellen bedingen. Apophysen als Ossifikationskerne in Sehnen- und Muskelinsertionen sind somit bei Heranwachsenden besonders vulnerabel für **überlastungsbedingte Pathologien**

überlastungsbedingte Pathologien.

Diese überlastungsbedingten Pathologien bzw. Apophysenschäden sind großteils sportassoziierte Veränderungen bei Kindern und Jugendlichen [10, 11, 12].

**Apophysenschäden**

Apophysenschäden

haben das Potenzial, meist ohne groß einschränkende Restzustände – wie veränderte Gelenkmechanik oder Längenunterschiede – durch Trainingspausen und konservative Therapie auszuheilen. Problematisch können jedoch unerkannte Apophysenverletzungen sein, da sie die sportliche Leistungsfähigkeit verändern und beim Jugendlichen nicht selten den Abbruch der Sportkarriere bedingen [4]. Prävention sowie Früherkennung durch gesicherte Diagnostik durch Ultraschall, Röntgen und Magnetresonanztomographie (MRT) helfen, dieser Fehlentwicklung entgegenzuwirken.

## Pathogenese und Diagnostik

Apophysen können als **sekundäre Ossifikationszentren**

sekundäre Ossifikationszentren

im Ansatzbereich von Sehnen verstanden werden. Sie treten in der zweiten Lebensdekade auf, um später mit dem angrenzenden Knochen zu fusionieren [13, 14]. Diese Ossifikationszentren sind mit einer Wachstumsknorpelfuge verbunden, die im Sinne einer Spina Tuber oder Tuberositas konfiguriert und bei Wachstumsabschluss ossifiziert. Anatomisch sind die epiphyseale Wachstumsplatte und die Apophyse ähnlich. Das zentrale Unterscheidungsmerkmal ist deren unterschiedliche Wachstumsrate [15]. Das Auftreten und die Fusion dieser Ossifikationszentren werden deshalb auch in die Wachstumsprognose als mögliche Parameter der biologischen Altersbestimmung herangezogen.

Apophysen sind per se ein **Locus minoris resistentiae**

Locus minoris resistentiae

[16]. Für diesen Umstand sind mehrere Mechanismen verantwortlich, wobei hier die 4 zentralen angeführt werden sollen:

1. Das Längenwachstum der Knochen übt eine hohe Zugspannung auf den umhüllenden Muskelmantel aus, der auch mit einer zunehmenden Dysbalance und vornehmlichen Verkürzung der gelenkübergreifenden Muskulatur vergesellschaftet ist.
2. Über eine vermehrte wachstumsassoziierte STH(somatotropes Hormon)-Ausschüttung tritt eine Verminderung der mechanischen Belastbarkeit auf [17].
3. Die einstrahlenden Kollagenfaserbündel der Sehnen werden durch den Ossifikationskern unterbrochen und umgelenkt. Somit verändert der Ossifikationskern im Sehnenansatz selbst auch die biomechanische Belastbarkeit des Sehnenansatzes.
4. Strukturen des heranwachsenden, muskuloskeletalen Systems können in einer „Hierarchie“ von Belastbarkeit verstanden werden. Dabei weist die Wachstumsknorpelzone der Apophyse und Epiphyse die geringste Belastbarkeit auf, gefolgt von Sehnen und Muskeln sowie Ligamenten, die am widerstandsfähigsten sind. Insofern zählen Apo- und Epiphysen zu den vulnerabelsten Strukturen bei Kindern und Jugendlichen [18].

### Cave

Apophysen gelten als „Locus minoris resistentiae“.

Dislokationen von Fragmenten sind als pathologische Folge von Belastung selten, da das straffe Periost und Perichondrium an der Sehneninsertionsstelle dies verhindert. Dennoch reißt manchmal der angrenzende Knochen mit aus. Diese Verletzungen der Apophyse zusammen mit **Fragmentdislokationen**

Fragmentdislokationen

werden als Apophysenfraktur oder Avulsion bezeichnet. Wegweisend in Richtung Indikation für die operative Therapie ist die mechanische Irritation durch ossäre Fragmente.

Im Vordergrund der Diagnostik für apophysäre Schäden stehen die klinische Präsentation und Anamnese des Patienten. Meist können Verdachtsdiagnosen durch ein zusätzliches, **konventionelles Röntgen**

konventionelles Röntgen

ausreichend spezifisch unterstützt werden. Das Ausmaß der Verletzungen variiert interindividuell stark und reicht von einer Apophysitis bis hin zu Frakturen im Sinne von Avulsionen.

Das **klinische Beschwerdebild**

klinische Beschwerdebild

zeigt sich zumeist unspezifisch mit Schmerzen und Schwellungen. Klassischerweise verstärkt passives Dehnen die Schmerzsymptomatik, wohingegen Ruhe diese Beschwerden vermindert. Obwohl das konventionelle Röntgen oftmals ausreichend spezifisch ist, muss dieses oft durch **Detailaufnahmen**

Detailaufnahmen

ergänzt werden. Diese richten sich nach der Lokalisation und werden durch Dreh- oder Detailaufnahmen ergänzt. Beispielsweise muss dies oft beim Verdacht auf Apophysenschäden des Trochanter minor gemacht werden, wobei das betroffene Bein im Röntgen oft rotiert werden muss, um eine möglichst eindeutige Darstellung der Struktur zu erreichen.

### Merke

Dehnen verschlimmert meist die Beschwerden betroffener PatientInnen.

### Cave

Das klinische Beschwerdebild zeigt sich zumeist unspezifisch.

Die **Sonographie**

Sonographie

kann bezogen auf die Sehnenbeteiligung helfen, den Zustand der Sehne und des knorpelig-knöchernen Überganges in der Apophyse zu beurteilen. Für die Darstellung der Vaskularisierung kann zusätzlich die Doppler-Technik helfen.

In manchen Fällen muss zusätzlich eine **MRT**

MRT

veranlasst werden. Dies trifft besonders zu, wenn der – angenommene – Apophysenschaden an untypischen Stellen wie dem Tuber ischiadicum vermutet wird. Hier ist die Differenzialdiagnose oft schwierig, da osteolytische Veränderungen auch an Tumoren denken lassen bzw. diese vortäuschen.

Richtungsgebend in der Diagnostik ist dennoch das Wissen um die häufigen Fehldiagnosen bzw. die nicht diagnostizierten oder verspätet diagnostizierten Apophysenschäden. Häufiger „pitfall“ ist deren Verwechslung mit Muskelverspannungen [19].

### Merke

Im Vordergrund der Diagnostik stehen die Klinik und die Anamnese.

## Sport und Apophysenschäden

Sport im Kindes- und Jugendalter führt zu vermehrten Belastungen der Apophysen. Dadurch ist diese Gruppe in Zusammenschau mit den oben beschriebenen pathophysiologischen Mechanismen prädisponiert, apophysäre Verletzungen zu erleiden. Besonders **chronisch-repetitive Belastungen**

chronisch-repetitive Belastungen,

die häufig in Überlastungen münden, führen zu diesen Pathologien. Apophysenschäden sind in dieser Altersgruppe mit ca. 16 % aller Verletzungen prominent vertreten [20]. Das Prädilektionsalter der Apophysenschädigung liegt zwischen dem 12. und 16. Lebensjahr. Jungen sind bis zu 9‑mal häufiger betroffen als Mädchen. Als Ursachen für diese geschlechtsspezifischen Unterschiede werden die vermehrte Muskelmasse bei Jungen, die unterschiedliche Hormonsituation in der Pubertät und die differierende, geschlechtstypische Sportausübung diskutiert [20, 21, 22].

Erwähnenswert bleibt, dass die **Zugspannungskräfte**

Zugspannungskräfte

auf die Sehnenansätze auch oft ohne sportliche Überbelastung zu Unregelmäßigkeiten bei der Ossifikation dieser Knochenkerne führen können. Bei sportlich nicht oder wenig aktiven Kindern und Jugendlichen Apophysenschäden gänzlich auszuschließen wäre somit falsch. Die einseitige Belastung des Muskelmantels durch Training und die Wiederholung von sportartspezifischen Bewegungsstereotypen sind jedoch die maßgeblich treibenden Faktoren. Somit treten manifeste Ossifikationsstörungen mit partieller Abhebung, Frakturierung oder Kondensierung der Apophysen auf. Auch einzelne Traumata mit punktuellen Belastungsspitzen und abrupter Muskelanspannung können neben der chronisch-repetitiven Belastung diese vulnerable Struktur an ihre Grenze hin zu Verletzungen im Sinne einer **Avulsion**

Avulsion

führen. Der klassische Verletzungsmechanismus betrifft zweigelenkige Muskeln: Dabei spannt ein Gelenk in Endstellung die Muskelgruppe vor, und eine ruckartige Streckbewegung des anderen Gelenks führt zum Schaden an den apophysären Sehnenansätzen. Ein augenscheinliches Beispiel dafür stellt der „Hürdenschritt“ dar. Dabei wird die Hüfte flektiert zusammen mit einer akzentuierten Streckbewegung im Kniegelenk.

### Cave

Chronisch-repetitive Belastungen führen häufig zu Überlastungen.

Beim ausgereiften Skelettsystem führt dieser Verletzungsmechanismus typischerweise zum Muskeleinriss. Beim heranwachsenden muskuloskeletalen System führt dies eher zu Schädigungen im Apophysenbereich der Sehne. Ein zentrales Thema im Zusammenhang mit intensiver Sportausübung im Kindes- und Jugendalter sind muskuläre Dysbalancen aufgrund von **sportartspezifischen Adaptationsphänomenen**

sportartspezifischen Adaptationsphänomenen

der Muskulatur mit wiederum sportartspezifischen Muskeldysbalancen. Typische Beispiele dafür sind ausgeprägte Psoas‑, Ischiocrural- und Rectus-femoris-Verkürzungen bei Fußballern, Handballern oder Tennisspielern.

**Figure 1:**
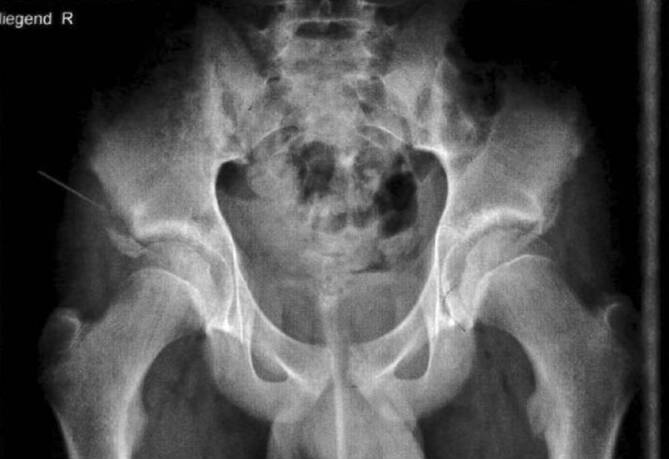

Abhängig vom sportartspezifischen Belastungsmuster treten die apophysären Schäden an typischen Lokalisationen auf. Beispielsweise zeigen sich bei Fußballern oder Basketballern, dem Belastungsmuster mit forcierten Kniestreck- und Hüftbeugebewegungen folgend, Apophysenstörungen im Bereich der Patellasehneninsertion an der Tuberositas tibiae wie beim Morbus Schlatter sowie im Bereich der Rectus-femoris-Insertion an der Spina iliaca anterior inferior häufig. Daher sind spezifische Typen von Apophysenverletzungen klassischerweise spezifischen Sportarten zuzuordnen. Bei Kohortenuntersuchungen mit Muskelfunktionstests von 12- bis 16-Jährigen konnten eindeutige **sportartspezifische Muskeldysbalancen**

sportartspezifische Muskeldysbalancen

festgestellt werden: Schwimmer zeigten kaum verkürzte Muskulatur an der unteren Extremität, während Ballsportler wie Fußball‑, Basketball‑, Volleyball- oder Tennisspieler hohe Raten verkürzter Muskeln zeigen. Die Häufigkeit von Knie- und Leistenschmerzen in diesen Sportarten unterstreicht die Bedeutung dieser hier oft überlasteten Strukturen [23]. Weitere Reihenuntersuchungen von 2567 Sportlern von Steinbrück et al. haben 57 Apophysenschäden am Becken festgestellt, 94 % davon betrafen Jungen. Die häufigste Sportart war Fußball, gefolgt von Leichtathletik (Sprint, Sprung). Die am häufigsten betroffenen Strukturen am Becken sind die Spina iliaca anterior superior mit dem Ansatz des M. tensor fascia latae und des M. satorius (Abb. 1), die Spina iliaca anterior inferior mit dem Rectusansatz des M. quadriceps, der Tuber ossis ischii mit dem Ansatz der ischiokruralen Muskeln, von Adduktorenanteilen und des M. quadratus femoris sowie der Trochanter minor mit dem Ansatz des M. iliopsoas [24].

Als mögliche Folgen nach apophysären Verletzungen werden unterschiedliche **Komplikationen**

Komplikationen

diskutiert. Darunter fallen Pseudarthrosen, Kallusformierungen, Fehldiagnosen u. v. m. Im Zentrum dieser Diskussionen steht aber die potenzielle Behinderung des Wachstums [25, 26, 27].

Differenzialdiagnostisch sollten sowohl Tumoren als auch Infektionen in Betracht gezogen werden [28].

Schlüssel für die erfolgreiche Behandlung bleibt die **frühe Diagnose**

frühe Diagnose.

Sobald bei jungen Athleten Schmerz auftritt, muss das Training sofort unterbrochen werden und ein Sportorthopäde bzw. Sporttraumatologe konsultiert werden. Wesentliche Säulen einer rationalen Präventionsstrategie von Apophysenschäden betreffen nicht nur einen Bereich, sondern müssen interdisziplinär und multiprofessionell umgesetzt werden, um nachhaltig erfolgreich zu sein. Das betrifft v. a. den ärztlichen Bereich, den Trainingsbereich als auch das soziale Umfeld.

## Apophysenschäden an der unteren Extremität

### Trochanter minor

Der M. iliopsoas zieht von der Wirbelsäule und dem Becken an das Femur und inseriert mit einer kräftig ausgebildeten Sehne am Trochanter minor des Femurs. Die Apophyse tritt dort um das 12. Lebensjahr im Sehnenansatz auf. Sobald eine **forcierte Hüftflexion**

forcierte Hüftflexion

gegen Widerstände geschieht wie bei Schussbewegungen, Sprints oder Abbremsmanövern im Laufzyklus im Rahmen verschiedener Ballsportarten wie Fußball, Basketball, Volleyball oder beim Laufen, entstehen ebendort Be- und Überlastungen. Diese Bewegung zählt zu den häufigsten Ursachen von Avulsionen in diesem Bereich [29]. Klinisch präsentiert sich der junge Sportler mit stechenden Schmerzen bei Bewegung in der Leistenregion, bei hinkendem Gangbild und flektierter Schonhaltung im Hüftgelenk. Bei der Diagnostik ist zu beachten, dass das Röntgenbild im **a.-p.-Strahlengang**

a.-p.-Strahlengang

etwas außenrotiert aufgenommen werden soll. Somit kommt die Apophyse besser zur Darstellung. Im Seitenvergleich fällt eine Verbreiterung auf, die manchmal mit einer ausgiebigen Dislokation des Ossifikationskerns kombiniert ist.

### Morbus Osgood-Schlatter

Eine repetitiv-chronische Überlastung und eine damit vergesellschaftete Avulsion der Apophyse an der Tuberositas tibiae wird in der Literatur als Morbus Osgood-Schlatter Literatur beschrieben [16, 30]. Die pathomorphologische Zuordnung zu den aseptischen Knochennekrosen ist veraltet und konnte durch neuere Untersuchungen nicht bestätigt werden. Vielmehr überbrückt der mächtige M. quadriceps femoris mit der Patellasehne das Kniegelenk, wobei durch das anteilsmäßig größte Wachstum der Epiphysenfuge des distalen Femurs ein enormer Zug auf die Sehneninsertion auftritt. Durch zusätzliche sportliche Belastung wie in Ball- oder Schlagsportarten kommt es zur chronischen Dislokation dieser Apophyse mit sekundären Ossifikationserscheinungen oder zum akuten Ausriss der Apophyse [31]. Diese **Ossikel**

Ossikel

in der ansatznahen Sehne sind relativ selten (<10 %) schmerzhaft und müssen nach Wachstumsabschluss entfernt werden. Das klinische Erscheinungsbild ist gekennzeichnet durch eine prominent und schmerzhaft auf Druck reagierende Tuberositas tibiae. Im Röntgen zeigen sich eine Anhebung und Verbreiterung der Apophyse mit oft unregelmäßigen Ossifikationen. Im Ultraschall ist oft eine Bursa unterhalb des Lig. patellae vor dem Hoffa zu erkennen, die vermehrt vaskularisiert und entzündlich verändert ist. Oft ist auch der eigentliche Schmerz ursächlich durch diese **Bursitis**

**Figure 2:**
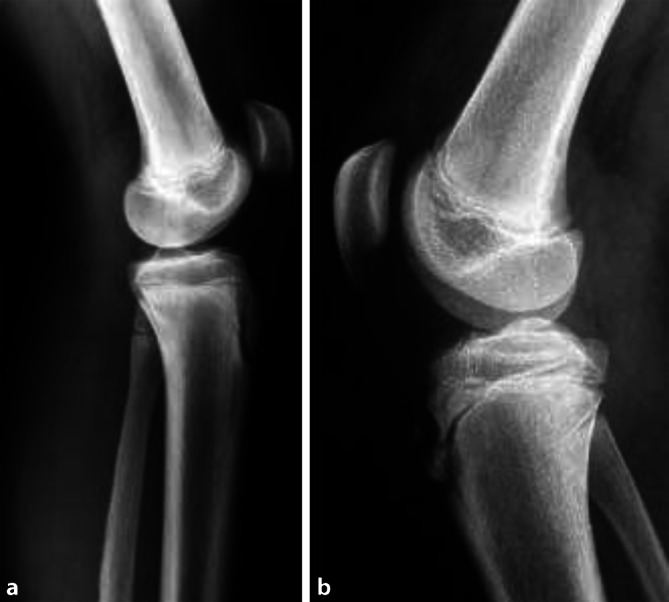

Bursitis

bedingt. Die knorpelig-knöcherne Übergangszone ist ähnlich einer Wachstumsfuge strukturiert und unregelmäßig konfiguriert. In schweren Fällen kann sich die gesamte Epiphyse angehoben und etwas nach dorsal disloziert präsentieren. Folge daraus ist ein Extensionsdefizit durch den vermehrten Slope. Als präventive Maßnahme ist besonders bei Ballsportarten wie Fußball oder Basketball [32] auf die Reifung der tibialen Epiphyse zu achten. Hier kann sich ein schnabelartiger Fortsatz in Richtung Tuberositas tibiae formen, der in weiterer Folge zu Wachstumsabschluss mit der Tibia fusioniert. Tritt eine Fragmentierung und Dislokation dieser Strukturen auf, ist eine Reduktion der Belastung dringend indiziert. Im Spätstadium liegen oft gerundete Ossikel in der Patellasehne, die Tuberositas erscheint verklumpt, gelegentlich auch ausgezipfelt (Abb. 2).

Als therapeutische Intervention sind Schonung, Kryotherapie und eine adaptierte, sportliche Aktivität anzuraten. Besonders die Reduktion von Bewegungsabläufen, die aus forcierten Endlagen strecken oder ähnliche Belastungsspitzen bedingen, ist zu vermeiden. Dies betrifft konkret Sprung- und Laufbelastungen. Auch kurzzeitige **Sportpausen**

Sportpausen

sind zentral. Das langfristige Ziel in Zusammenarbeit mit der Physiotherapie muss die Wiederherstellung der Muskelbalance der Oberschenkelmuskulatur sein. Bei Persistenz der Beschwerden und Ausbildung eines intratendinösen Ossikels muss die Exzision des Knochenfragments angedacht werden [33, 34, 35].

### Morbus Sinding-Larsen-Johansson

Der Morbus Sinding-Larsen-Johansson ist vom Morbus Osgood-Schlatter abzugrenzen. Beide Erkrankungen stehen im Zusammenhang mit dem Streckapparat des Knies bei Kindern und Jugendlichen. Jedoch wird hier ein Schmerzsyndrom am proximalen Patellasehnenansatz, der mit einer tendinösen Überlastung der Insertionsstelle am distalen Patellapol einhergeht, beschrieben.

Diese Erkrankung kann – aus einer pragmatisch-klinischen Sicht – als „jumpers knee“ bezeichnet werden. Die Tendinose besteht hier am **Patellapol**

Patellapol

mit intratendinösen Sehnenveränderungen. Diese zeigt sich mit Überlastungsnekrose oder Partialrupturen. Folge davon sind oft kalkdichte, schollige Veränderungen am distalen Patellapol. Typisch sind die Druckschmerzhaftigkeit des distalen Patellapols [36] sowie Signalalterationen des tendinoossären Überganges im Ultraschall und in der MRT.

### Morbus Sever

Schmerzhafte Überlastungen der **Kalkaneusapophyse**

**Figure 3:**
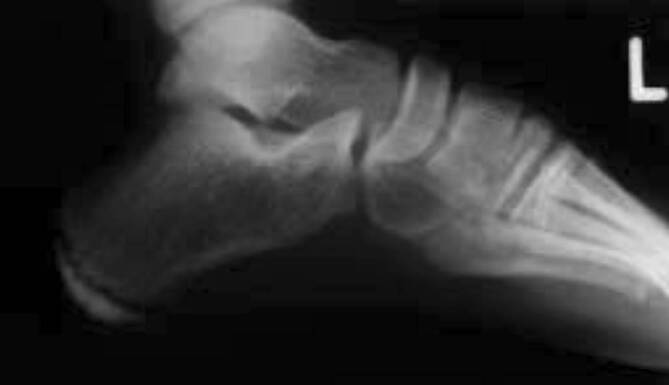

**Figure 4:**
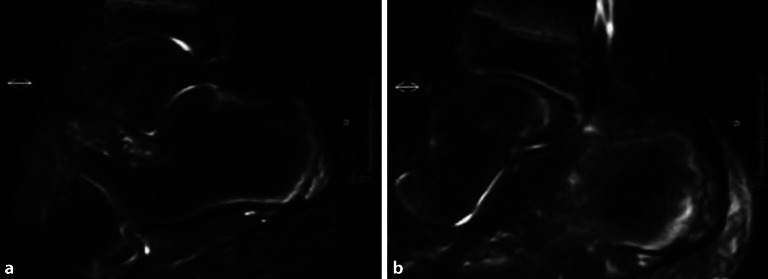

Kalkaneusapophyse

werden als Morbus Sever bezeichnet. Der Morbus Sever tritt gehäuft bei fußbetonten Sportarten wie Basketball oder Fußball, aber auch im Lauf- und Sprungsport auf [30, 37]. Klassischerweise präsentieren sich betroffene Patienten mit einem Druckschmerz der Ferse von plantar und dorsal unterhalb des Achillessehnenansatzes. Im Röntgen erscheint die Apophyse verbreitert, gelegentlich fragmentiert und kondensiert (Abb. 3). In der Diagnostik muss bedacht werden, dass auch die Apophyse bei Fersen von Gesunden sehr vielgestaltig ausgeformt sein kann. Dieser Umstand erschwert oft eine genaue Zuordnung. In der zusätzlichen MRT (Abb. 4a, b) ist daher meist ein Knochenödem im Bereich der fragmentierten Apophyse sichtbar und wegweisend. Therapeutisch im Vordergrund stehen wieder die **konservative Behandlung**

konservative Behandlung

mit Einlagenversorgung und Schuhzurichtungen mit Fersendämpfung sowie das Dehnen der Wadenmuskulatur.

## Apophysenschäden an der oberen Extremität

In Summe sind Apophysenschäden an der oberen Extremität seltener verglichen mit der unteren Extremität. Dies kann unter anderem dadurch erklärt werden, dass die am häufigsten ausgeübten Sportarten wie Fußball oder Basketball primär die untere Extremität belasten.

### Ellenbogen – „little league ellbow“

Apophysenschäden am medialen Epikondylus des Ellenbogens werden als „little league ellbow“ bezeichnet. Davon betroffen sind typischerweise junge Baseballspieler. Die chronisch-repetitiven **Wurfbewegungen**

Wurfbewegungen

zusammen mit dem **Valgusstress**

Valgusstress

während der Beschleunigungsphase scheinen ätiopathologisch im Zentrum zu stehen.

Hang et al. fanden in einer retrospektiven Studie, dass 70 % der Fänger und 63 % der Werfer unter Little-League-Spielern eine Separierung des medialen Epikondyls aufwiesen. Von diesen waren 56 % bzw. 49 % symptomatisch [38].

Der berühmte Sportorthopäde Lyle Micheli [39] hat das Problem des Kindersports sehr prägnant dargestellt: „too much, too soon“.

## Fazit für die Praxis

- Zentrale Ursache für Apophysenschäden stellen Trainingsfehler mit zu hohen Belastungen zu einem biologisch zu frühen Zeitpunkt dar.
- Die Apophysen sind als Locus minoris resistentiae in der Muskel-Sehnen-Knochen-Kette das schwächste Glied und können bei übermäßiger Beanspruchung oder abrupten Belastungen im Sinne von akuten und chronischen Avulsionen geschädigt werden.
- Zentrale Maßnahmen in der Prävention sind eine systematische Trainingsplanung und die Vermeidung mechanischer Überbeanspruchung im Wachstumsalter.
- Eine exakte Bildgebung ermöglicht die Diagnose von Frühstadien apophysärer Strukturschäden, die in dieser Phase durch Trainingspause und konservative Maßnahmen geheilt werden können.
- Das Fortsetzten des Trainings bei Apophysenschäden führt zu ausgeprägten Verformungen und Umbauprozessen an den Apophysen, die oft nachhaltig die Belastbarkeit des Bewegungsapparates beeinflussen.
- Sportorthopädische Probleme im Kindesalter sind in vielen Fällen vermeidbar.

### Merke

Too much, too soon.

## CME-Fragebogen

Welche ist eine der häufigsten Fehldiagnosen im Falle apophysärer Verletzungen?

Fraktur

Muskeleinriss

Knorpelfraktur

Muskelverspannung

Tendinopathie

Welcher Risikofaktor beschreibt am treffendsten den pathophysiologischen Mechanismus für apophysäre Verletzungen?

Chronisch-repetitiv

Zyklisch-repetitiv

Explosiv

Isometrisch

Statisch

Welche Hierarchie des heranwachsenden muskuloskeletalen Systems bezogen auf Belastbarkeit – beginnend mit der schwächsten – ist korrekt? (Muster: schwächste Struktur < … < stärkste Struktur)

Apophysen/Epiphysen < Sehnen < Muskeln < Ligament

Sehnen < Apophysen/Epiphysen < Muskeln < Ligament

Apophysen/Epiphysen < Ligament < Sehnen < Muskeln

Apophysen/Epiphysen < Muskeln < Sehnen < Ligament

Ligament < Muskel < Sehnen < Apophysen/Epiphysen

Wie viel Prozent stellen in etwa die apophysären Verletzungen in der Altersgruppe von Kindern und Jugendlichen dar?

50 %

23 %

34 %

90 %

16 %

Zu welcher Verletzung führt typischerweise der Pathomechanismus der Apophysenverletzung (Gelenk in Endstellung mit vorgespannter Muskelgruppe – ruckartige Streckbewegung des anderen Gelenks) bei Erwachsenen?

Ligamentruptur

Knorpelfraktur

Knochenfraktur

Muskeleinriss

Typischerweise keine Verletzung

Wie ist das Verhältnis von apophysären Verletzungen bezogen auf das Geschlecht (m:f)?

9:1

8:1

7:1

6:1

5:1

Welche First-line-Therapie ist bei den meisten apophysären Verletzungen nötig und oft ausreichend?

Physiotherapie

Muskuläre Stärkung

Trainingspause + Trainingsplanadaptation

Dehnungsübungen

Operative Sanierung

Welche anatomische Struktur ist beim „little league ellbow“ betroffen?

Medialer Epikondylus

Lateraler Epikondylus

Caput radii

Olekranon

Tuberositas radii

Ein junger Sportler (männlich, 12. Lebensjahr) präsentiert sich in Ihrer Sprechstunde mit folgendem Symptombild: stechender Leistenschmerz bei Bewegung, hinkend, flektierte Schonhaltung. Für den potenziellen Fall einer apophysären Verletzung denken Sie an welche Struktur?

Trochanter minor

Trochanter major

Tuberositas tibiae

Kalkaneus

Tuber ischiadicum

Ein junger Sportler (männlich, 13. Lebensjahr) präsentiert sich mit Schmerzen in der Region des proximalen Unterschenkels bzw. distal der Patella. Sie denken an einem Morbus Osgood Schlatter. Bei der klinischen Untersuchung zeigt sich der Hauptschmerzpunkt jedoch eher am distalen Patellapol. An welche Erkrankung denken Sie differenzialdiagnostisch?

Morbus Sever

Morbus Sinding-Larsen-Johansson

Morbus Haglund

Morbus Perthes

Morbus Renander
